# Do Swedish rock-climbers exhibit more eating disorder and body dissatisfaction symptoms than non-climbers? A cross-sectional study

**DOI:** 10.1136/bmjopen-2024-085265

**Published:** 2024-10-16

**Authors:** Isabel Nigicser, Fredrik Identeg, Mikael Sansone, Henrik Hedelin, Niklas Forsberg, Ulrika Tranaeus, Klara Edlund

**Affiliations:** 1 Department of Orthopedics, Institute of Clinical Sciences, Sahlgrenska Academy, University of Gothenburg, Gothenburg, Sweden; 2 Department of Orthopaedics, NU-Hospital Group, Trollhättan/Uddevalla, Sweden; 3 Department of Physiology, Nutrition, Biomechanics; Sport Performance & Exercise Research & Innovation Center - Stockholm, SPERIC-S; The Swedish School of Sport and Health Sciences, Stockholm, Sweden; 4 Institute of Environmental Medicine, Karolinska Institutet, Stockholm, Sweden; 5 Division of Psychology, Department of Clinical Neuroscience, Karolinska Institutet, Stockholm, Sweden

**Keywords:** mental health, sports medicine, psychiatry

## Abstract

**Objectives:**

The inclusion of rock-climbing in the Olympic Games has increased participation in the sport and attention to athletes' health. In sports where the importance of low body weight is considerate, there is an increased risk of developing eating disorders (EDs). There is sparse research on EDs among rock-climbers. The primary aim was to map ED symptoms among rock-climbers compared with controls. The secondary aim was to analyze differences in body dissatisfaction. Comparisons between rock-climbing levels and sexes were performed.

**Design:**

This is a cross-sectional study in a larger prospective longitudinal study series, CLIMB: Climber’s Longitudinal attitudes towards Injury, Mental health and Body image, using baseline data.

**Participants:**

Swedish rock-climbers, at an advanced and elite level according to the International Rock Climbing Research Association, participated. Participants were at least 13 years old. 183 rock-climbers partook with a mean age of 29.5 (SD=9.1) with two participants under 15 years old. 180 controls partook with a mean age of 29.0 (SD=8.9). There were no control participants under 15 years of age.

**Primary and secondary outcome measures:**

Data was gathered through a self-report questionnaire collecting the primary outcome measure, ED symptoms (Eating Disorders Examination Questionnaire (EDE-Q) V.6.0) and the secondary outcome measure, body dissatisfaction (Body Shape Questionnaire-8C).

**Results:**

There were no differences between rock-climbers and controls regarding ED symptoms. Symptoms were higher among female rock-climbers (26%) than male (5.8%). Regarding body dissatisfaction, the control group reported higher levels compared with rock-climbers. At least a mild concern was observed in 13.3% of male rock-climbers, compared with 47.4% of females.

**Conclusions:**

Although there was no difference in the EDE-Q V.6.0 between rock-climbers and controls, females reported significantly more symptoms than men. Regarding body dissatisfaction, female rock-climbers exhibited higher levels of dissatisfaction than males. Further, higher levels of dissatisfaction were reported in controls, especially in men, where symptoms were three times higher than rock-climbers.

**Trial registration number:**

NCT05587270.

**Strengths and limitations of this study**

- The use of validated questionnaires as well as the inclusion of a control group.
- The large population of Swedish rock-climbers group that the authors believe accurately represents the general climbing population in Sweden.
- One limitation is the use of a self-selected sample, not allowing for a randomised sample.
- Another limitation is the use of self-report questionnaires only.

## Introduction

 The recent inclusion of rock-climbing in the Olympic Games has led to increased participation in the sport and subsequent attention to the athlete’s health. A body mass index (BMI) score below 18.5 kg/m^2^ is classified as underweight and has been linked to health problems in sports as well as in the general population. According to Bratland-Sanda and colleagues, eating disorder (ED) prevalence ranges from 6% to 45% in female athletes and up to 19% in male athletes.^1^ In sports where the importance of low body weight is considerate, athletes who compete appear to be particularly vulnerable to ED development.^2^ Disordered eating (DE) is defined as a spectrum between optimised nutrition and EDs.^3^ Athletes may fluctuate along this spectrum during their career, and DE has been considered a risk factor for developing EDs.^4^ The International Federation of Sport Climbing as recently as January 2024, detailed the physical consequences of EDs among climbers and the need for preventing unhealthy athletes from competing in the 2024 Olympics in Paris.

Sports are commonly classified as ‘lean’ or ‘non-lean’ based on the association between low body weight and increased performance, with rock-climbing falling into the ‘lean’ category. Participants of ‘lean’ sports are at an increased risk of developing pathological eating behaviours in order to achieve better performance.^5 6^ It has been observed that ED symptoms in elite athletes range from 37% to 60%, with athletes employing measures such as using laxatives, vomiting, diuretics and binge eating before competitions.^7^,^9^ According to a meta study by Mancine *et al*, ED prevalence is higher among elite athletes and only a small number seek treatment.^10^ Female’s scores were higher than male’s, indicating that differences between sexes must be considered.^11^

Adolescents are especially vulnerable and often employ unhealthy methods for weight loss.^12^ DE behaviours, including restrictive dieting, binge eating and unhealthy weight control practices, are more prevalent in adolescents than full-blown EDs. Research indicates that 20–30% of adolescents engage in some form of DE. DE behaviours are also prevalent among adults, with estimates suggesting that 10–20% engage in such behaviours. These behaviours may either persist from adolescence or develop later in life. Thus, adolescence is a critical period for the onset of EDs like anorexia and bulimia, with early diagnosis and intervention crucial for improving outcomes.^13^ In adults, EDs are often more chronic, leading to a higher burden of illness over time.^14^ Further, EDs are more common in females in both adolescents and adults, although males are increasingly recognised as being at risk for DE behaviours. EDs and DE are prevalent concerns across both age groups, though they manifest differently in adolescents and adults. Adolescents are at higher risk for the development of these disorders, while adults often face more chronic conditions.^15^ Understanding these distinctions is essential for effective prevention, diagnosis and treatment strategies.^16^

Regarding the link between rock climbing and EDs, research is lacking. To date, only a small number of publications exist measuring symptoms of DE in climbers, and these have examined the occurrence of ED only in high-level competing athletes without comparison to a control group. Studies examining the occurrence of ED symptoms in climbers of lower levels, and studies of longitudinal design are lacking.^17^ A recent qualitative study in rock-climbers has shown that EDs are a problem relevant to the climbing community, and that it is a dominant idea that a low weight is beneficial.^18^ Regarding injury, a 2023 study reports that the OR for having an injury doubled for those rock-climbers with an ED.^19^ Another recent publication on elite rock-climbers report higher levels of amenorrhoea among those with EDs.^20^ With the increased number of athletes in rock-climbing and the risk for decreased health due to EDs, it is important to conduct further studies in this area.

The primary aim of this study was to compare the prevalence of ED symptoms among rock-climbers versus controls. Secondly, body dissatisfaction was examined among rock-climbers versus controls. Differences between climbing levels (advanced vs elite/higher elite) as well as between sexes were examined. The authors main hypothesis was that elite and advanced rock-climbers would present more DE symptoms compared with controls, while elite rock-climbers were hypothesised to show more symptoms than advanced rock-climbers. This is due to the current asumption that elite rock-climbers are employing higher levels of weight control strategies in order to achieve higher performance compared with advanced climbers.

## Methods

### Participants

Inclusion criteria were based on the International Rock Climbing Research Association (IRCRA) grading tool: Male rock-climbers must have climbed a boulder route rated harder than 6B (non-flash ascent) or a lead route (red-point ascent) rated 7a+ within the last year. Females must have climbed a boulder route rated harder than 6A or a lead route rated 6c within the last year. There were no exclusion criteria other than that participants had to be at least 13 years of age.

The control group consisted primarily of university students recruited via posted signs in the university buildings. The control group was matched for number and were non-rock-climbers who had not competed in any sport at the elite level and who were not training more than 3 days/week. All participants were at least 13 years of age.

### Procedure

This is a cross-sectional analysis of baseline data from the CLIMB study, which is a 2-year longitudinal project.^21^ Swedish rock-climbers at the advanced, elite and higher elite levels according to the IRCRA^22^ were invited to participate in the study. Information was distributed by the Swedish Climbing Federation via climbing gyms, clubs and national teams. The study was also promoted on social media channels such as Facebook and Instagram. Participants provided written consent before inclusion. This study is approved by the Swedish Ethical Review Authority (2021-05557-01).

### Patient and public involvement

None.

### Study measures

Data collection was performed using a self-reported web-based survey. Demographics were reported as biological sex, age and height and weight used to calculate BMI. To capture the level of the rock-climbers, IRCRA’s grading scales were applied. Rock-climbers were then grouped into two groups: advanced rock-climbers, and elite and higher elite rock-climbers. An additional table (online supplemental table 1) is included detailing descriptive data regarding the climber’s opinions on the connections between body weight and appearance and performance. The protocol for the present cross-sectional study provides further detail on design.^21^

### Primary variable

#### Eating Disorder Examination Questionnaire

DE was measured by the validated Eating Disorders Examination Questionnaire (EDE-Q V.6.0), V.6.0^23^ with a Swedish language version also demonstrating satisfactory levels of internal consistency in adolescents between 13 and 15 years of age compared with adults.^24^ The EDE-Q is composed of 28 items with four primary subscales: (1) Restraint eating: Assesses dietary restraint, including the intention to restrict food intake to influence shape or weight and the avoidance of foods thought to be fattening. Example item: ‘Have you been deliberately trying to limit the amount of food you eat to influence your shape or weight (whether or not you have succeeded)?’, (2) Eating concern: Measures preoccupation with food, eating and eating-related anxiety, including guilt about eating and secrecy surrounding eating behaviour. Example item: ‘Has thinking about food, eating, or calories made it very difficult to concentrate on things you are interested in (such as work, hobbies, and so on)?’, (3) Shape concern: Focuses on the importance placed on body shape, dissatisfaction with body shape and fear of gaining weight. This subscale is highly indicative of body image disturbance. Example item: ‘Have you had a definite desire to have a totally flat stomach?’, (4) Weight concern: Evaluates concern about body weight, including the fear of weight gain, dissatisfaction with current weight and the importance of weight in self-evaluation. Example item: ‘How dissatisfied have you been with your weight?’ The EDE-Q also provides a global score, which is the average of the four subscale scores. This global score gives an overall indication of the severity of ED pathology. A global cut-off score of >2.5 was used as indicative of subclinical ED and >4.0 for serious clinical ED pathology in agreement with earlier publications.^25^,^28^ The EDE-Q has demonstrated adequate test–retest reliability (r=0.89) and internal consistency (α=0.85).^29^

### Secondary variable

#### Body Shape Questionnaire

To gain insight into the participant’s body dissatisfaction, the valid Swedish version of the short 8-item version of the Body Shape Questionnaire (BSQ-8C)^30^,^32^ was employed, which has been shown to have high internal consistency (α=0.94) as well as excellent test–retest properties.^33^ The BSQ-8C is comprised of 8 items to which the participants respond on a 6-point Likert-like scale from ‘never’ to ‘always’. Example items are, ‘Has feeling full (eg, after eating a large meal) made you feel fat?’, and ‘Have you imagined cutting off fleshy areas of your body?’. A score under 19 indicates no concern with shape. A score from 19 to 25 indicated a mild concern with shape. A score from 26 to 33 indicates a moderate concern with shape, and a score over 33 indicates marked concerns with shape.

### Statistical methods

Data was analysed using SAS software V.9.4 (SAS Institute, North Carolina, USA). For categorical variables, n (%) is presented. Nominal values are presented as n (%). For continuous variables mean (SD) is presented when normally distributed. When skewed, median and IQR is presented (IQR). For comparison between groups, Fisher’s Exact test was used for dichotomous variables and the Mantel-Haenszel χ^2^ Exact test was used for ordered categorical variables and the Fisher’s non-parametric permutation test was used for continuous variables. Threshold for statistical significance was set at p<0.05.

There is sparse existing data regarding DE among rock-rock-climbers. However, based on previous retroactive data from injuries in rock-climbers,^34^ using a significance level of 5%, a power of 80% and an expected relative risk of 1.5 for the primary outcome, the present study calculated that approximately 55 participants would be needed in both the climbing group and the control group.

## Results

### Subject characteristics

As displayed in table 1, there were a total of 183 rock-climbers and 180 controls. One climber failed to report climbing levels completed in the past 3 months and was therefore excluded from analyses where grouping based on climbing level was used. Among rock-climbers, 57.4% were male and 42.6% were female. Among controls, 39.4% were male and 60.6% female, yielding a significant difference in sex distribution. The mean age of 29.5 in rock-climbers (SD=9.1) with two adolescents under 15 years of age, and 29.0 (SD=8.9) among controls, with no adolescents under 15 years of age. In rock-climbers, the mean BMI was 22.1 (SD=2.3) and 23.3 (SD=4.1) in controls (p=<0.01). Among rock-climbers, 20 participants (11%) were under 18 years of age. Among controls, 1 participant (<0.01) was under 18 years of age. A further age breakdown of participants is supplied in online supplemental table 2. Table 2 details the training habits of the climbing groups with respect to years of experience, number of training sessions per week and number of hours per session.

**Table 1:** The distribution of study participants and subject characteristics

Demographics	Climber (n=183)	Control (n=180)	P value
Male (n, (%))	105 (57.4)	71 (39.4)	
Female (n, (%))	78 (42.6)	109 (60.6)	<0.01
Age (years, (SD))	29.5 (9.1)	29.0 (8.9)	0.6
BMI (kg/m^2^, (SD))	22.1 (2.3)	23.3 (4.1)	<0.01

BMIbody mass index

**Table 2:** Rock climbers’ characteristics and training habits

	Advanced (n=135)			Elite/higher elite (n=47)		
	Total	Men	Woman	Total	Men	Women
Age (years, (SD))	30.8 (9.0)	32 (9)	29 (8)	25.8 (8.4)	26 (9)	26 (8)
Height (cm, (SD))	175 (9)	180 (6)	167 (7)	171 (11)	179 (9)	164 (8)
Weight (kg, (SD))	69 (10)	74 (7)	61 (8)	63 (13)	72 (11)	54 (7)
BMI (kg/m^2^, (SD))	22.4 (2.2)	22.9 (2.1)	21.7 (2.2)	21.2 (2.1)	22.2 (2.0)	20.2 (1.8)
Years rock-climbing (n, (IQR))	4 (3;9)	5 (2;10)	4 (3;7)	8 (5;12)	8 (6;12)	9 (5;14)
Rock-climbing sessions per week (n, (SD))	3 (1)	3 (1)	3 (1)	4 (1)	4 (1)	4 (1)
Rock-climbing hours per session (n, (IQR))	2 (2;3)	2 (2;3)	3 (2;3)	3 (2;3)	3 (2;3)	3 (2;4)

BMIbody mass index

### Primary variable outcome, the EDE-Q V.6.0 global score

There was no statistical difference between the rock-climbers and controls regarding the EDE-Q V.6.0 global score (p=0.2), see table 3. Among rock-climbers, 14.2% have at least a subclinical indication of an ED compared with 22.2% in the control group. At least a subclinical ED indication was exhibited in 5.7% of the males, whereas this was present in 26% of females (p<0.01), see table 4. Additionally, in the control group, 15.5% of males reported at least a subclinical ED indication compared with 26.7% of females. Among rock-climbers, at least a subclinical indication of ED was present in 14.1% in advanced group and 14.9% in the groups of elite and higher elite rock-climbers, see table 5.

**Table 3:** Results for ED and body dissatisfaction for rock climbers versus controls

	Climber (n=183)	Control (n=180)	P value
EDE-Q V.6.0 global score (n (%))			
No ED indication	157 (85.8)	140 (77.8)	
Subclinical indication of ED	20 (10.9)	36 (20.0)	
Serious ED clinical indication	6 (3.3)	4 (2.2)	0.2
Percentage with at least a subclinical indication of ED	14.2	22.2	
BSQ-8C (n (%))			
No concern with shape	132 (72.1)	107 (59.4)	
Mild concern with shape	21 (11.5)	31 (17.2)	
Moderate concern with shape	21 (11.5)	18 (10.0)	
Marked concerns with shape	9 (4.9)	24 (13.3)	<0.01
Percentage with at least mild concerns with shape	27.9	40.5	

For comparison between groups Fisher’s Exact test (lowest one-sided p value multiplied by 2) was used for dichotomous variables. The Mantel-Haenszel χ2 Exact test was used for ordered categorical variables. The Mantel-Haenszel χ2 test was used for ordered categorical variables. The Fisher’s non-parametric permutation test was used for continuous variables.

BSQ-8CBody Shape Questionnaire-8CEDeating disorderEDE-QEating Disorder Examination Questionnaire

**Table 4:** Outcomes between male and female rock-climbers

	Males (n=105)	Females (n=77)	P value
EDE-Q V.6.0 global score (n (%))			
No ED indication	99 (94.3)	57 (74.0)	
Subclinical indication of ED	5 (4.8)	15 (19.0)	
Serious ED clinical indication	1 (1.0)	5 (6.5)	<0.01
Percentage with at least a subclinical indication of ED	5.7	26.0	
BSQ-8C (n (%))			
No concern with shape	91 (86.7)	41 (53.2)	
Mild concern with shape	8 (7.6)	12 (15.6)	
Moderate concern with shape	4 (3.8)	17 (22.1)	
Marked concerns with shape	2 (1.9)	7 (9.1)	<0.01
Percentage with at least mild concerns with shape	13.3	47.4	

For comparison between groups Fisher’s Exact test (lowest one-sided p value multiplied by 2) was used for dichotomous variables. The Mantel-Haenszel χ2 Exact test was used for ordered categorical variables. The Mantel-Haenszel χ2 test was used for ordered categorical variables. The Fisher’s non-parametric permutation test was used for continuous variables.

BSQBody Shape QuestionnaireEDeating disorderEDE-QEating Disorder Examination Questionnaire

**Table 5:** Advanced rock-climbers versus elite rock-climbers, n=182

	Advanced (n=135)	Elite/higher elite (n=47)	P value
EDE-Q V.6.0 global score (n (%))			
No ED indication	116 (85.9)	40 (85.1)	
Subclinical indication of ED	16 (11.9)	4 (8.5)	
Serious ED clinical indication	3 (2.2)	3 (6.4)	0.5
Percentage with at least a subclinical indication of ED	14.1	14.9	
BSQ-8C (n (%))			
No concern with shape	98 (72.6)	34 (72.3)	
Mild concern with shape	13 (9.6)	7 (14.9)	
Moderate concern with shape	19 (14.1)	2 (4.3)	
Marked concerns with shape	5 (3.7)	4 (8.5)	1.00
Percentage with at least mild concerns with shape	27.4	27.7	

For comparison between groups Fisher’s Exact test (lowest one-sided p value multiplied by 2) was used for dichotomous variables. The Mantel-Haenszel χ2 Exact test was used for ordered categorical variables. The Mantel-Haenszel χ2 test was used for ordered categorical variables. The Fisher’s non-parametric permutation test was used for continuous variables.

BSQBody Shape QuestionnaireEDE-QEating Disorder Examination Questionnaire

### Secondary variable outcome, BSQ-8C

At least a mild concern with shape was seen among 27.9% of rock-climbers, compared with 40.5% among the control group (p<0.01; table 3). At least a mild concern was observed in 13.3% of male rock-climbers, compared with 47.4% of their female counterparts (p<0.01). 7 of the 77 female rock-climbers (9.1%) reported having marked concerns with their shape and 2 of the 105 males reported this level of dissatisfaction (1.9%), see table 4. There was no statistical difference between advanced versus elite and higher elite rock-climbers (table 5).

### Rock-climbers’ perceptions on body weight importance

To better understand rock-climber’s own perceptions on body weight and performance as well as body ideals, they were asked to report how much they agree with the statements in online supplemental table 1. Of all rock-climbers, 76% at least partially agreed with the first statement that keeping a low body weight plays a large role in performance. Further breakdown is: 82% of all advanced rock-climbers, 80% of elite/higher elite rock-climbers, 83% of males and 67% of all females.

Of all rock-climbers, 84% at least partially agreed with the second statement that keeping a light and well-defined body is considered to be ideal in the sport. Further breakdown is: 83% of all advanced rock-climbers, 85% of elite rock-climbers, 85% of males and 82% of females.

## Discussion

The main results of this study displayed the high prevalence of clinically relevant ED symptoms and body dissatisfaction among rock-climbers, similar to levels reported in other lean sports. Although the primary variable (EDE-Q V.6.0 global score) rendered no difference between rock-climbers and controls nor between the two climbing levels, almost five times as many female rock-climbers experienced symptoms of body shape concern compared with their male counterparts, which is also in line with previous research in lean sports. A surprising result was that among males, control participants reported almost three times the amount of ED symptoms as the rock-climbers. The novelty of research on the mental health and eating habits of rock-climbers renders this study to be of significance and value, as it contributes to the foundation of knowledge in a growing sport. Understanding how rock-climbers compare and contrast with other athletes is therefore of increasing importance.

Existing meta-analyses and systematic reviews on self-reported disordered eating (SRDE) have thus far not included rock-climbers. However, trends can be observed. For example, a 2024 meta-analysis among athletes worldwide concluded that female sex, older age and higher BMI (all p<0.01) are associated with higher prevalence rates of SRDE.^35^ In a systematic review focused on DE prevalence in athletes categorised by leanness, six out of seven studies had a significant increase in DE rates among lean sport types.^5^ A 2022 meta-analysis on female athletes versus controls reported, like the current study, lower levels of body dissatisfaction in athletes than controls. It also concluded that DE behaviour was more prevalent in lean sports.^36^ The current study showed similar results, and including rock-climbing in future meta-analysis will provide a clearer comparison.

There are some recent, although few publications that have examined the link between climbing and DE. The results of Michael *et al*^37^ indicated no difference in ED symptoms between males and females.^37^ This contrasts the present study, which demonstrated a much higher reported level among females compared with males, both with regards to the ED global scale and body dissatisfaction. This is also reported in the study by Kristjánsdóttir, where moderate tosevere body dissatisfaction was almost six times higher among females than males.^11^ This is comparable to the present study, with symptoms being five times higher in females than males.

Rock-climbers reported less body dissatisfaction than controls. A possible explanation is that rock-climbers may feel that they get the exercise they require and therefore do not compensate with extra exercise to control their weight, whereas control participants may have the compulsion to train to make up for their lack of exercise or to fulfil social ideals. Edlund *et al*^38^ examined the link between body dissatisfaction, compulsive exercise and depression among university students and revealed that 38% reported at least mild concern with their shape. This is similar to this study’s result of 40.5% in the control group. In Edlund *et al*’s further comparison between sexes, females reported more body dissatisfaction, also in line with the present study.^38^

Elite athletes not only must cope with general societal body ideals, but also experience pressure in their sport-specific environments to be lean.^39^ Despite this added pressure, this study suggested that ED symptoms are not significantly higher than controls. It was found among Spanish elite athletes in 2020 that they strive for a body which is able to excel athletically, but also that is perfectly aligned with the societal ideal.^40^ This phenomenon was recently researched in a 2022 qualitative study among six rock-climbers, four of which were females, whose interviews confirmed the social norm that female rock-climbers should have a lean appearance and that DE was a normalised part of climbing.^41^

The practical implications of this study can be partly highlighted in online supplemental table 1. Rock-climbers reported their opinions on the connection between body weight and performance as well as the perceived ideal body type. 76% reported that they at least partially agree with the first statement that ‘keeping a low body weight plays a large role in performance’. A staggering 84% of rock-climbers at least partially agreed with the second statement that ‘keeping a light and well-defined body is considered to be the ideal within the sport’. This high level of agreement is in line with the 2023 qualitative study, emphasising the need for in-depth interviews. An increased awareness among coaches and health professionals will help to identify and prevent further development of EDs and DE in rock-climbers. Another aspect to consider is seasonal variation and the effect on the climbing seasons. The Swedish Scandinavian winter climate ranges from mild in the south to a subarctic climate in the north,^42^ rendering outdoor climbing much more impractical than indoor gym climbing. It would therefore be beneficial to observe variations in rock-climbers’ indoor versus outdoor habits in countries with different climates, and see the effects on the above-mentioned norms and ED symptoms.

The large scope of variables tested is a prominent strength, as research among rock-climbers is new and quickly rising. The participants of this study come from several cities in Sweden, which provides a range of age and ability that the authors believe accurately represent the general climbing population in Sweden. The use of validated questionnaires is a strength, as well as the inclusion of a control group. A limitation of this study is the use of a self-selected sample, not allowing for a randomised sample. Also, it has been shown that elite athletes tend to under-report ED symptoms in studies,^43^ which indicates that the level of ED symptoms could be underestimated. A limitation among the climbers is that there were only 47 elite participants, rendering the elite group underpowered. The present study must therefore consider the potential effects of type 2 errors and cannot conclude that elite rock-climbers do not have a higher level of ED symptoms compared with advanced rock-climbers. Survivorship bias must also be considered; could it be that some rock-climbers have such high levels of ED symptoms that they are unable to climb at an elite level? This would place them at a lower climbing level and thereby skew results.

### Conclusions

It may be concluded from the present study that although there was no difference in the EDE-Q V.6.0 global score between rock-climbers and controls, female rock-climbers reported more ED symptoms compared with males. Regarding the secondary variable body dissatisfaction, females rock-climbers exhibited higher levels of dissatisfaction compared with males. More symptoms were reported in the control group compared with rock-climbers. There was no difference between the advanced and elite climbing groups. Future research will benefit from follow-up measurements to identify and explore ED and DE trends in rock-climbers, as well as comparing between countries with different social norms and climates.

## supplementary material

10.1136/bmjopen-2024-085265 online supplemental file 1

10.1136/bmjopen-2024-085265 online supplemental file 2

## Data Availability

No data are available.
