# Correction for ‘Do swedish rock-climbers exhibit more eating disorder and body dissatisfaction symptoms than non-climbers? A crosssectional study’

**DOI:** 10.1136/bmjopen-2024-085265corr1

**Published:** 2025-03-05

Nigicser I, Identeg F, Sansone M, *et al*. Do Swedish rock-climbers exhibit more eating disorder and body dissatisfaction symptoms than non-climbers? A crosssectional study. *BMJ Open* 2024;14:e085265. doi:10.1136/bmjopen-2024–0 85 265.

This article has been corrected since it was published online. The correction is regarding a value in table 4 regarding levels of body dissatisfaction. In the Female column, it says that the percentage with at least a mild concern with shape is 29.1%. This is incorrect, and it should instead read 47.4%. It is stated correctly in the Results portion of the paper, but the value in the table is incorrect.

