# Supplementary material for: Do Swedish rock-climbers exhibit more eating disorder and body dissatisfaction symptoms than non-climbers? A cross-sectional study
Source: BMJ Open. 2024 Oct 16;14(10):e085265. doi: 10.1136/bmjopen-2024-085265 (PMC11488074; doi:10.1136/bmjopen-2024-085265)
Supplement: online supplemental file 1 [file bmjopen-14-10-s001.pdf]

Supplementary Table 1: Rock-climbers' perceptions on body weight importance.

		Total Male and Female Rock-Climbers			
		Advanced		Elite/Higher Elite	
		Male Count n (%)	Female Count n (%)	Male Count n (%)	Female Count n (%)
**Keeping a low body weight plays a large role in how well one can perform in climbing.**	Completely Agree	6 (7)	4 (8)	4 (19)	0 (0)
	Mostly agree	16 (19)	7 (13)	2 (10)	5 (25)
	Partially agree	47 (56)	23 (43)	12 (57)	10 (50)
	Mostly don't agree	14 (17)	13 (25)	1 (5)	4 (20)
	Don't agree at all	1 (1)	6 (11)	2 (10)	1 (5)
**Keeping your body light and well-defined in considered to be the ideal body in climbing.**	Completely agree	10 (12)	10 (19)	6 (29)	5 (25)
	Mostly agree	27 (32)	14 (26)	6 (29)	6 (30)
	Partially agree	34 (40)	19 (36)	6 (29)	6 (30)
	Mostly don't agree	11 (13)	7 (13)	1 (5)	2 (10)
	Don't agree at all	2 (2)	3 (6)	2 (10)	1 (5)
**Due to the fact that it is considered to be the ideal body within the sport.**	Completely agree	11 (13)	9 (17)	5 (24)	4 (20)
	Mostly agree	17 (20)	12 (23)	4 (19)	3 (15)
	Partially agree	28 (33)	22 (42)	4 (19)	8 (40)
	Mostly don't agree	22 (26)	4 (8)	3 (14)	1 (5)
	Don't agree at all	6 (7)	6 (11)	5 (24)	4 (20)
**As a climber it is important to control your weight in order to perform well.**	Completely agree	5 (6)	1 (2)	5 (24)	1 (5)
	Mostly agree	14 (17)	11 (21)	7 (33)	1 (5)
	Partially agree	37 (44)	19 (36)	6 (29)	9 (45)
	Mostly don't agree	23 (27)	12 (23)	2 (10)	5 (25)
	Don't agree at all	5 (6)	10 (19)	1 (5)	4 (20)
