# Supplementary material for: Do Swedish rock-climbers exhibit more eating disorder and body dissatisfaction symptoms than non-climbers? A cross-sectional study
Source: BMJ Open. 2024 Oct 16;14(10):e085265. doi: 10.1136/bmjopen-2024-085265 (PMC11488074; doi:10.1136/bmjopen-2024-085265)
Supplement: online supplemental file 2 [file bmjopen-14-10-s002.pdf]

Supplementary Table 2: Age breakdown among rock-climbers vs controls.

		Rock-Climbers vs Controls											
		Climber						Control					
		man		woman		Total		man		woman		Total	
		Count	N (%)	Count	N (%)	Count	N (%)	Count	N (%)	Count	N (%)	Count	N (%)
**Age groups**	13-14	2	1.9	0	0.0	2	1.1	0	0.0	0	0.0	0	0.0
	15-17	8	7.6	10	12.8	18	9.8	0	0.0	1	0.9	1	0.6
	18-25	23	21.9	20	25.6	43	23.5	32	45.1	50	45.9	82	45.6
	25-30	26	24.8	19	24.4	45	24.6	22	31.0	22	20.2	44	24.4
	31-40	28	26.7	24	30.8	52	28.4	13	18.3	16	14.7	29	16.1
	41+	18	17.1	5	6.4	23	12.6	4	5.6	20	18.3	24	13.3
	Total	105	100.0	78	100.0%	183	100.0	71	100.0	109	100.0	180	100.0
