# Supplementary material for: Do Swedish rock-climbers exhibit more eating disorder and body dissatisfaction symptoms than non-climbers? A cross-sectional study
Source: BMJ Open. 2024 Oct 16;14(10):e085265. doi: 10.1136/bmjopen-2024-085265 (PMC11488074; doi:10.1136/bmjopen-2024-085265)
Supplement: Supplementary file 3 [file bmjopen-2024-085265.reviewer_comments.pdf]

PEER REVIEW HISTORY

BMJ Open publishes all reviews undertaken for accepted manuscripts. Reviewers are asked to complete a checklist review form and are provided with free text boxes to elaborate on their assessment. These free text comments are reproduced below.

ARTICLE DETAILS

Title (Provisional)

Do Swedish rock-climbers exhibit more eating disorder and body dissatisfaction symptoms than non-climbers? A cross-sectional study.

Authors

Nigicser, Isabel; Identeg, Fredrik; Sansone, Mikael; Hedelin, Henrik; Forsberg, Niklas; Tranaeus, Ulrika; Edlund, Klara

---

VERSION 1 - REVIEW

**Reviewer**	**1**
**Name**	**Mohammadi, Shooka**
**Affiliation Department**	**ACECR, Integrative Oncology and Quality of life**
**Date**	**14-Mar-2024**
**COI**	**No**

• Please replace all instances of "we" or "our" with phrases such as "current study," "this study," or "present study".

• The manuscript lacks a clear identification of the specific gap it intends to address, and does not introduce any novel insights to the field in comparison to existing literature. The rationale of the study should be explained better. The necessity of such research should be more highlighted.

• First paragraph of introduction is too long! (Should not be longer than 15 lines).

• Some sentences are too long and decrease the readability. They should be shorter.

• Extensive paraphrasing and proofreading are required. The quality of writing is not good.

• The discussion should be more concise and include more references for better comparison with previous research.

• Strengths and limitations of study should be explained at the end of discussion.

Conclusion section also should be modified.

**Reviewer**	**2**
**Name**	**Jankauskienė, Rasa**
**Affiliation**	**Lithuanian Sports University**
**Date**	**25-Mar-2024**
**COI**	**I have no competing interests.**

Thank you for the possibility to review this manuscript. Eating disorders (ED) and disordered eating (DE) are serious body image-related issues that might negatively affect the health and performance of athletes. The prevalence of DE and ED is understudied and the study adds important new knowledge to the topic. The study aimed to assess the prevalence of DE symptoms among rock climbers and to compare them with controls. Further, the manuscript aimed to examine and compare body dissatisfaction between rock climbers and controls. Next, the authors aimed to compare DE symptoms between sports levels of rock climbers and between sexes. The sample consisted of 183 rock – climbers with a mean age of 29,5 years, and 180 controls (mean age of 29 years). There were found no differences between rock climbers and controls, however, DE was more prevalent in women compared to men and body dissatisfaction was more prevalent in controls compared to rock climbers. No differences were observed between the sports levels of rock climbers.

Here are my comments that might help to increase the quality of the manuscript:

Abstract

1) The mean age, age range, and number of sex in separate groups of the sample is lacking.

2) I do not agree that the design of the study was prospective (line 42).

3) Conclusions should be revised to generalize the results of the study.

Introduction

1) The introduction gives the reader some insight into the problem, however, I would recommend rewriting it to present the differences between ED and DE in adolescents (boys and girls), adults (women and men), and in groups of different sports levels of rock climbing. Please clearly state what is known about the prevalence of ED and DE separately in these groups.

2) Please do not mix findings related to the prevalence of DE and ED in different groups of sex, age, and sports mastery.

3) I would appreciate it if the aim of the study would be more concentrated and focused on the main idea. Was it a comparison of DE between rock climbers and controls or between sexes? Looking at the name of the manuscript seems that the main aim is to compare ED between sexes, however, the aims of the study are different.

4) The Introduction lacks hypotheses that should be stated after the aims of the study.

Methods

1) Please divide information between the Participants and Procedure and develop the Procedure subsection. For example, information in line 31, page 5 does not suit here.

2) The authors state that there were no exclusion criteria. Was it possible that that participants younger than 13 years to participate in the study?

3) Please give reference to the Procedure where it is possible to find the full protocol of the study.

4) Please clearly state what was the number of adolescents and adults in your sample and provide this information for controls.

5) The instruments are not presented normally. Please provide a full description of the instruments with examples of possible answers, validation of instruments in athletes and referencing for validation of instruments in the Swedish language.

6) Please also provide references for the cut-offs for subclinical and clinical scores for athletes.

7) Is EDEQ-6 a suitable measure for athletes at the age of 13 years?

8) I have some doubts that BSQ measures body dissatisfaction. Please present the instrument normally.

9) Please insert the text in lines 26-33, p.6 into the Participants subsection.

Results

1) The tables do not suit the requirements for the scientific journals, please check the requirements.

2) The names of the tables should be revised.

3) The results must be presented according to the revised aims of the study. I recommend presenting firstly information comparing rock climbers and controls, then women and men, and then between sports levels groups. Please clarify what is the main aim of the study and present results accordingly.

4) Please also indicate the statistical considerations reporting the differences between groups in the text.

Discussion

1) Please start the discussion with the main aim and hypotheses and then clearly state what novelty the present study adds.

2) Please avoid discussing the strengths and weaknesses of the study in the second paragraph of the Discussion. This should be done at the end of the section.

3) Please do not go to the details of sample size power calculations in the third paragraph, This information should be included in Methods, not in Discussion.

4) Please organize the Discussion focusing on the main aims and previous findings and indicating if it is related to the prevalence of DE or ED.

5) There is nothing new that ED and DE are more prevalent in women and body dissatisfaction is higher in the general population compared to athletes. There is are lot of literature on this topic, please address this literature, especially systematic reviews.

6) Please develop a paragraph discussing the practical implications of the study.

7) Please revise the Conclusions using generalized statements about main findings.

What is already known on the topic

1) ED are prevalent in women compared to men in all sports.

2) The third sentence seems confusing. You should discuss what is already known on the topic.

What this study adds:

1) Please do not mix rock, climbers, men, women, and controls. Please clearly state what you mean according the study aims.

VERSION 1 - AUTHOR RESPONSE

Dear editorial board,

We thank the reviewers for their comments and thorough feedback. We have taken all comments into account and appreciate how this has improved the manuscript. We have addressed the reviewer's feedback below point by point.

To assist in readability of the revisions, we have specified the lines edited when applicable and referred to where in the text the changed were made.

Please see the attached file with point-by-point reponses.

Best regards,

Isabel Nigicser

Corresponding author

Reviewer comment:

Please ensure that your abstract is formatted according to our Instructions for Authors: http://bmjopen.bmj.com/pages/authors/#research

Author response:

The authors have reviewed the Instructions and have adjusted to ensure formatting compliance.

Reviewer comment:

Please complete a thorough proofread of the text and correct any spelling and grammar errors that you identify, for example, “In sports where the importance of low body weight is considerate, there is an icreased risk of developing eating disorders (EDs).” and “Athletes may display DE behaviors such as skipping meals, using diuretics, engage in compulsive excercise, all while not meeting the criteria for ED [3].” and “The control group was matched fin number and were non-rock-climbers who had not competed in any sport at the elite level and who were not training more than three days/week.” and “It may be concluded from the present study that although there was no difference in the EDE-Q 6.0 global score between rock-climbers and controls nor between the climbing groups butfemale rockclimbers reported more ED symptoms compared to males.”

Author response:

A thorough proofreading of the text has been completed and spelling and grammar errors have been corrected.

Reviewer comment:

Please remove the ‘What is already known on this topic, What this study adds’ section, as this does not conform to BMJ Open’s formatting guidelines.

Author response:

These sections have now been removed.

Reviewer comment:

Along with your revised manuscript, please include a copy of the STROBE checklist indicating the page/line numbers of your manuscript where the relevant information can be found (https://strobe-statement.org/index.php?id=strobe-home).

Author response:

A copy of the STROBE checklist is included.

Reviewer comment:

Please provide a statement clarifying how participants provided consent (ie., written/ verbal).

Author response:

A statement clarifying consent has been added to the first paragraph in the “Participants” section of the study.

Reviewer comment:

Formatting Amendments (where applicable).

Author response:

The authors have attempted to adjust any formatting incongruencies according to the requirements.

Reviewer 1:

Reviewer comment:

Please replace all instances of "we" or "our" with phrases such as "current study," "this study," or "present study".

Author response:

Instances of “we” or “our” have been replaced with alternative phrases or removed altogether where possible.

Reviewer comment:

The manuscript lacks a clear identification of the specific gap it intends to address, and does not introduce any novel insights to the field in comparison to existing literature. The rationale of the study should be explained better. The necessity of such research should be more highlighted.

Author response: A clarification of the scientific gap, the study aims to fill has been added in the introduction (page 3, line 5-14)

Reviewer comment:

First paragraph of introduction is too long! (Should not be longer than 15 lines).

Author response:

The first introduction paragraph has been shortened and is now 12.5 lines.

Reviewer comment:

Some sentences are too long and decrease the readability. They should be shorter.

Author response:

The authors have proofread and shortened sentences in the paper to increase readability, especially in the Introduction and Discussion.

Reviewer comment:

Extensive paraphrasing and proofreading are required. The quality of writing is not good.

Author response:

The authors have proofread and paraphrased portions of the paper with superfluous information, aiming to make the manuscript more succinct.

Reviewer comment:

The discussion should be more concise and include more references for better comparison with previous research.

Author response:

The Discussion is now more concise and includes a new paragraph with three recent systematic reviews/Meta-analyses for better comparison with research on this topic.

Reviewer comment:

Strengths and limitations of study should be explained at the end of discussion.

Author response:

According to the above link for formatting http://bmjopen.bmj.com/pages/authors/#research, after "Supplementary and raw data", the strengths and limitations come earlier in the discussion. However, the authors have adjusted the order in the discussion to adhere to the reviewer's comment.

Reviewer comment:

Conclusion section also should be modified.

Author response:

The authors have attempted to use generalized statements which answer the study's aims, avoiding too much information while presenting overall conclusions.

Reviewer 2:

Comments to the Author:

Thank you for the possibility to review this manuscript. Eating disorders (ED) and disordered eating (DE) are serious body image-related issues that might negatively affect the health and performance of athletes. The prevalence of DE and ED is understudied, and the study adds important new knowledge to the topic. The study aimed to assess the prevalence of DE symptoms among rock climbers and to compare them with controls. Further, the manuscript aimed to examine and compare body dissatisfaction between rock climbers and controls. Next, the authors aimed to compare DE symptoms between sports levels of rock climbers and between sexes. The sample consisted of 183 rock – climbers with a mean age of 29,5 years, and 180 controls (mean age of 29 years). There were found no differences between rock climbers and controls, however, DE was more prevalent in women compared to men and body dissatisfaction was more prevalent in controls compared to rock climbers. No differences were observed between the sports levels of rock climbers.

Here are my comments that might help to increase the quality of the manuscript:

Abstract

Reviewer comment:

The mean age, age range, and number of sex in separate groups of the sample is lacking.

Author response:

The mean age, age range, and number of climbers and controls are now in the Participants section.

Reviewer comment:

I do not agree that the design of the study was prospective (line 42).

Author response:

The authors have clarified the meaning of the original sentence. The current study is cross-sectional, using baseline data from a larger prospective study (CLIMB) planning to continue for 2 years.

Reviewer comment:

Conclusions should be revised to generalize the results of the study.

Author response:

The authors have attempted to use generalized statements, avoiding too much information while presenting overall conclusions.

Introduction

Reviewer comment:

The introduction gives the reader some insight into the problem, however, I would recommend rewriting it to present the differences between ED and DE in adolescents (boys and girls), adults (women and men), and in groups of different sports levels of rock climbing. Please clearly state what is known about the prevalence of ED and DE separately in these groups.

Author response:

The authors thank you for the recommendation. We have added to the manuscript.

Reviewer comment:

Please do not mix findings related to the prevalence of DE and ED in different groups of sex, age, and sports mastery.

Author response:

The authors have removed information to make these portions of the introduction more succinct, they now include only generalized results which help the reader understand the current existing studies.

Reviewer comment:

I would appreciate it if the aim of the study would be more concentrated and focused on the main idea. Was it a comparison of DE between rock climbers and controls or between sexes? Looking at the name of the manuscript seems that the main aim is to compare ED between sexes, however, the aims of the study are different.

Author response:

The reviewer raises a good point. The aim of the study was to compare DE between rock climbers and controls. The differences between sexes were also examined and presented as they yielded noteworthy results. Regarding the name of the manuscript, the authors have changed it to better reflect the aims of the study.

Reviewer comment:

The Introduction lacks hypotheses that should be stated after the aims of the study.

Author response:

The author's hypothesis is now included after the aims of the study.

Methods

Reviewer comment:

Please divide information between the Participants and Procedure and develop the Procedure subsection. For example, information in line 31, page 5 does not suit here.

Author response:

The authors have looked at page 5 line 31, which is information regarding Subject Characteristics in the Results section of the paper. It is therefore somewhat difficult to discern what the reviewer is referring to, as there is no particular subsection entitled "Procedure". The authors have attempted to include all relevant information required in the Material and Methods section of the paper.

Reviewer comment:

The authors state that there were no exclusion criteria. Was it possible that that participants younger than 13 years to participate in the study?

Author response:

The authors have revised the sentence in question to include that participants must be at least 13 years of age.

Reviewer comment:

Please give reference to the Procedure where it is possible to find the full protocol of the study.

Author response:

The authors have included a reference under "Design and Measurements" to the full study protocol.

Reviewer comment:

Please clearly state what was the number of adolescents and adults in your sample and provide this information for controls.

Author response:

The authors have added this information in the "Subject characteristics" portion of the "Results", as this is thought by the authors to be the more appropriate portion since it could not be determined ahead of time how many adolescents will participate. An Adolescent is defined as participants under the age of 15.

Reviewer comment:

The instruments are not presented normally. Please provide a full description of the instruments with examples of possible answers, validation of instruments in athletes and referencing for validation of instruments in the Swedish language.

Author response:

The authors have added references and example questions to supplement the presented instruments.

Reviewer comment:

Please also provide references for the cut-offs for subclinical and clinical scores for athletes.

Author response:

The references are provided.

Reviewer comment:

Is EDEQ-6 a suitable measure for athletes at the age of 13 years?

Author response:

Thank you for the question, the authors have added a reference regarding the satisfactory levels of internal consistency in adolescents.

Reviewer comment:

I have some doubts that BSQ measures body dissatisfaction. Please present the instrument normally.

Author response:

The authors have added an example question to help clarify this matter and explained the cutoffs which represent levels of concern with shape.

Reviewer comment:

Please insert the text in lines 26-33, p.6 into the Participants subsection.

Author response:

Thank you for the suggestion. The authors assume the reviewer is referring to the Subject Characteristics subsection of the Results. As it is impossible to predict the exact group demographics, the authors deemed that this information was rather better fitted to the Results section than the Material and Methods section. The participant requirements, as well as inclusion and exclusion criteria, are presented appropriately in the Material and Methods section.

Results

Reviewer comment:

The tables do not suit the requirements for the scientific journals, please check the requirements.

Author response:

The authors have adjusted the table formats according to our best knowledge. They are “copy” and “pasted” from a separate Excel document, but are not embedded.

Reviewer comment:

The names of the tables should be revised.

Author response:

The names of the tables have been revised to include a description on how data is presented, for example n(%) or mean (SD).

Reviewer comment:

The results must be presented according to the revised aims of the study. I recommend presenting firstly information comparing rock climbers and controls, then women and men, and then between sports levels groups. Please clarify what is the main aim of the study and present results accordingly.

Author response:

The authors have presented the results according to the reviewer's recommendation above, in accordance with the clarified aims of the study.

Reviewer comment:

Please also indicate the statistical considerations reporting the differences between groups in the text.

Author response:

The authors unfortunately do not understand the meaning of the request, and would like to kindly ask for clarification. Statistical methods are reported in the Materials and Methods section. If the reviewer is referring to the tables in the text, the titles of these have been changed to better reflect the nature of the data being presented.

Discussion

Reviewer comment:

Please start the discussion with the main aim and hypotheses and then clearly state what novelty the present study adds.

Author response:

The authors have now started the discussion accordingly.

Reviewer comment:

Please avoid discussing the strengths and weaknesses of the study in the second paragraph of the Discussion. This should be done at the end of the section.

Author response:

According to the above link for formatting http://bmjopen.bmj.com/pages/authors/#research, after "Supplementary and raw data", the strengths and limitations come earlier in the discussion. However, the authors have adjusted the order in the discussion to adhere to the reviewer's feedback.

Reviewer comment:

Please do not go to the details of sample size power calculations in the third paragraph, This information should be included in Methods, not in Discussion.

Author response:

The authors have adjusted the third paragraph (now the last paragraph of the Discussion) accordingly.

Reviewer comment:

Please organize the Discussion focusing on the main aims and previous findings and indicating if it is related to the prevalence of DE or ED.

Author response:

The Discussion is now reorganized to focus on main aims as recommended above.

Reviewer comment:

There is nothing new that ED and DE are more prevalent in women and body dissatisfaction is higher in the general population compared to athletes. There is are lot of literature on this topic, please address this literature, especially systematic reviews.

Author response:

The Discussion now includes a paragraph with three recent systematic reviews/meta-analyses on this topic.

Reviewer comment:

Please develop a paragraph discussing the practical implications of the study.

Author response:

The authors have now reworked and adjusted the discussing addressing the practical implications of the study in the 6th paragraph of the Discussion.

Reviewer comment:

Please revise the Conclusions using generalized statements about main findings.

Author response:

The authors have attempted to use generalized statements, avoiding too much information while presenting overall conclusions.

What is already known on the topic

Reviewer comment:

ED are prevalent in women compared to men in all sports.

Author response:

This section of the paper has been removed as it does not comply with formatting regulations.

Reviewer comment:

The third sentence seems confusing. You should discuss what is already known on the topic.

Author response:

This section of the paper has been removed as it does not comply with formatting regulations.

What this study adds

Reviewer comment:

Please do not mix rock, climbers, men, women, and controls. Please clearly state what you mean according the study aims.

Author response:

This section of the paper has been removed as it does not comply with formatting regulations.

VERSION 2 - REVIEW

**Reviewer**	**2**
**Name**	**Jankauskienė, Rasa**
**Affiliation**	**Lithuanian Sports University**
**Date**	**18-Jun-2024**
**COI**	**No competing interests**

The authors addressed some of my comments and I believe the manuscript's quality was increased. However, some issues need the additional attention of the authors:

The abstract should report the mean age and standard deviation (SD is lacking). Also, the clear number and percentage of adolescents in the main and control groups separately should be reported. A significant difference in sex distribution was observed between climbers and control groups. Maybe a significant difference in the numbers of adolescents and adults exists between these groups?

Two adolescents were under 15 years of age. How many adolescents were between 15 and 17 years of age? What proportion of adolescents were in both samples? Was this proportion equal? Adolescence involves the period from 11 to 18 years.

The introduction was rewritten. However, a discussion about the prevalence of DE and ED in adolescent and adult samples is needed. Also, the prevalence of DE and ED in elite and competitive athletes (adolescents and adults) differs. I recommend the authors do a better job of including this information in the Introduction. Now the Introduction seems eclectic.

Also, I would appreciate it if the authors gave a better rationale in the Introduction for their hypothesis of the study. For example, it is difficult to understand why elite rock climbers should report more symptoms of ED compared to advanced climbers.

Also, I disagree, that „to date, only a small number of publications exist measuring symptoms of ED“. A plethora of studies did that.

Methods

Please develop the Procedure (Study Design) section and divide it between the Participants and Procedure subsections. The participant's subsection describes the procedure rather than providing information about participants. Also, the subsection „Design and Measurements“ should be renamed to the Instruments of the Study or Study Measures.

The EDE-Q 6.0 is still not presented properly. Please provide the item examples of all subscales and also please explain to the reader what possible answers are. Also please provide information on what the higher score means. Please also provide the reference for cut-offs. Also, please give information if the EDE-Q 6 is validated for use in athletes. There are some specific scales developed for screening athletes and EDEQ was developed for use in the general population. Why do authors use EDE-Q 6 rather than specific instruments for testing DE in athletes? Please also address this as the limitation of the study in the Discussion.

Please also use the same remarks regarding the presentation of the Body Shape Questionnaire. The author of the BSQ questionnaire is missing.

Results

The authors used the Fisher Exact test and Chi-Square tests to compare groups, however, the values of these tests are not presented in the text of the Results section or in tables. Please provide this information.

The authors revised the names of the Tables. The name of Table 1 is „Demographic data and subject characteristics. Data is presented as mean (SD)“. Please revise it as „Table 1. The distribution of study participants and subject characteristics“. Please rename first column variables as (male (n, percentage), women (n, percentage), age (years, n (SD) and BMI (kg/m2). Please revise all names and Tables accordingly. Use notes instead of explanations in the names of Tables. Avoid the sign % in all lines of tables if the name of Tables already says that it is a percentage.

The discussion is now of better quality. However, I still would like to see a clear statement in the first paragraph explaining to the reader what new this study adds. Please also make clear comparisons of the prevalence of DE and ED with previous studies in lean sports and your study. So, was the prevalence in DE and ED higher compared to other samples of lean sports or similar? The reader needs clear answers in the first paragraph of the Discussion.

VERSION 2 - AUTHOR RESPONSE

Dear editorial board,

We thank the reviewer for their comments and thorough feedback on this revised paper. We have taken all comments into account and appreciate how this has improved the manuscript. We have addressed the reviewer's feedback below point by point.

To assist in readability of the revisions, we have referred to where in the text the changed were made. Please see the attached file.

Best regards,

Isabel Nigicser

Reviewer comment:

The abstract should report the mean age and standard deviation (SD is lacking). Also, the clear number and percentage of adolescents in the main and control groups separately should be reported. A significant difference in sex distribution was observed between climbers and control groups. Maybe a significant difference in the numbers of adolescents and adults exists between these groups?

Author response:

The standard deviation has been added to the abstract. More data about the number of adolescents has been added to the "Subject Characteristics" portion of the Results, as the authors believe this information fits better there than the abstract. A further breakdown of age data is supplied in Supplementary table 1.

Reviewer comment:

Two adolescents were under 15 years of age. How many adolescents were between 15 and 17 years of age? What proportion of adolescents were in both samples? Was this proportion equal? Adolescence involves the period from 11 to 18 years.

Author response:

A further breakdown of age data is supplied in the newly added Supplementary table 1.

Reviewer comment:

The introduction was rewritten. However, a discussion about the prevalence of DE and ED in adolescent and adult samples is needed. Also, the prevalence of DE and ED in elite and competitive athletes (adolescents and adults) differs. I recommend the authors do a better job of including this information in the Introduction. Now the Introduction seems eclectic. Also, I would appreciate it if the authors gave a better rationale in the Introduction for their hypothesis of the study. For example, it is difficult to understand why elite rock climbers should report more symptoms of ED compared to advanced climbers.

Author response:

The authors thank the reviewer for the feedback. More information regarding the prevalence of ED and DE in adolescents vs adults has been added to the introduction (paragraph 3), as well as a better rationale for the hypothesis for the study.

Reviewer comment:

Also, I disagree, that „to date, only a small number of publications exist measuring symptoms of ED". A plethora of studies did that.

Author response:

The authors should have been clearer in this statement, it was intended to convey that there are only a small number of publications on ED in rock-climbers, instead of athletes in general. A clarification has been added to the above quoted sentence.

Methods

Reviewer comment:

Please develop the Procedure (Study Design) section and divide it between the Participants and Procedure subsections. The participant's subsection describes the procedure rather than providing information about participants. Also, the subsection „Design and Measurements" should be renamed to the Instruments of the Study or Study Measures.

Author response:

The authors have divided the Participants section and moved procedure information to a new paragraph titled "Procedure". Also, the "Design and Measurements" section has been renamed to "Study Measures", according to the reviewer's recommendation.

Reviewer comment:

The EDE-Q 6.0 is still not presented properly. Please provide the item examples of all subscales and also please explain to the reader what possible answers are. Also please provide information on what the higher score means. Please also provide the reference for cut-offs. Also, please give information if the EDE-Q 6 is validated for use in athletes. There are some specific scales developed for screening athletes and EDEQ was developed for use in the general population. Why do authors use EDE-Q 6 rather than specific instruments for testing DE in athletes? Please also address this as the limitation of the study in the Discussion.

Author response:

Further details on item examples have been added according to the reviewer's recommendation, as well as a clarification on what the higher score means. References for the cut-offs have been added. Information on EDE-Q in athletes has been added.

The EDE-Q is sensitive enough to detect subclinical symptoms, making it an essential tool for understanding the nuanced relationship between sport, body image, and eating behaviors in this population. Athletes, especially those in weight sensitive sports often exhibit subclinical eating disorder symptoms that might not meet full diagnostic criteria but still significantly impact health and performance (e.g., Sundgot-Borgen & Torstveit, 2010). Also, The EDE-Q can be adapted or interpreted with consideration of sport-specific risk factors, such as pressures related to body weight and shape, which are prevalent among athletes. This makes it a relevant tool for capturing the specific eating-related challenges faced by athletes in both weight-sensitive and endurance sports (e.g., Martinsen & Sundgot-Borgen, 2013).

Higher scores on the EDE-Q are indicative of more severe eating disorder symptoms.

Further, since we are also assessing a sample from the general population the EDE-Q was considered the best fit for our study where we address a sample with both athlete- and general populations. We therefore, respectfully, view the use of the EDE-Q as an appropriate tool, and is not addressed as a limitation.

Relevant references:

Fairburn, C. G., & Beglin, S. J. (1994). "Assessment of eating disorders: Interview or self-report questionnaire?" International Journal of Eating Disorders, 16(4), 363-370.

Mond, J. M., Hay, P. J., Rodgers, B., & Owen, C. (2006). "Eating Disorder Examination Questionnaire (EDE-Q): Norms for young adult women." Behaviour Research and Therapy, 44(1), 53-62.

Luce, K. H., & Crowther, J. H. (1999). "The reliability of the Eating Disorder Examination-Self-Report Questionnaire version (EDE-Q)." International Journal of Eating Disorders, 25(3), 349-351.

Berg, K. C., Peterson, C. B., Frazier, P., & Crow, S. J. (2012). "Psychometric evaluation of the Eating Disorder Examination and Eating Disorder Examination-Questionnaire: A systematic review of the literature." International Journal of Eating Disorders, 45(3), 428-438

Martinsen, M., & Sundgot-Borgen, J. (2013). Higher prevalence of eating disorders among adolescent elite athletes than controls. Medicine and Science in Sports and Exercise, 45(6), 1188-1197.

Sundgot-Borgen, J., & Torstveit, M. K. (2010). Aspects of disordered eating continuum in elite high-intensity sports. Scandinavian Journal of Medicine & Science in Sports, 20(2), 112-121.

Reviewer comment:

Please also use the same remarks regarding the presentation of the Body Shape Questionnaire. The author of the BSQ questionnaire is missing.

Author response:

Further details on item examples have been added as well as the author of the BSQ-8c.

Results

Reviewer comment:

The authors used the Fisher Exact test and Chi-Square tests to compare groups, however, the values of these tests are not presented in the text of the Results section or in tables. Please provide this information.

Author response:

The values of these tests are now added in tables 3-5.

Reviewer comment:

The authors revised the names of the Tables. The name of Table 1 is „Demographic data and subject characteristics. Data is presented as mean (SD)“. Please revise it as „Table 1. The distribution of study participants and subject characteristics“. Please rename first column variables as (male (n, percentage), women (n, percentage), age (years, n (SD) and BMI (kg/m2). Please revise all names and Tables accordingly. Use notes instead of explanations in the names of Tables. Avoid the sign % in all lines of tables if the name of Tables already says that it is a percentage.

Author response:

The tables’ titles and content have been modified. Explanations are no longer in the table titles, and notes have been added to the first columns, so that all percentage signs could be removed.

Reviewer comment:

The discussion is now of better quality. However, I still would like to see a clear statement in the first paragraph explaining to the reader what new this study adds. Please also make clear comparisons of the prevalence of DE and ED with previous studies in lean sports and your study. So, was the prevalence in DE and ED higher compared to other samples of lean sports or similar? The reader needs clear answers in the first paragraph of the Discussion.

Author response:

The authors thank the reviewer for their feedback. A statement at the end of the first paragraph has been added explaining what this study adds (the last two sentences). In paragraph 2 and 3 the authors make clear comparisons between our study and other lean sports, addressing ED and DE.
